# Diagnosis and management of *de novo* inflammatory bowel disease after solid organ transplantation in the era of biologic therapy: a case series

**DOI:** 10.3389/frtra.2024.1483943

**Published:** 2025-01-08

**Authors:** Willie Mohammed Johnson, Byron P. Vaughn, Nicholas Lim

**Affiliations:** ^1^ Department of Medicine, University of Minnesota, Minneapolis, MN, United States; ^2^ Division of Gastroenterology, Hepatology and Nutrition, University of Minnesota, Minneapolis, MN, United States

**Keywords:** solid organ transplant, Crohn's disease, ulcerative colitis, immunosuppression, inflammatory bowel disease

## Abstract

**Introduction:**

The clinical characteristics of *de novo* inflammatory bowel disease (dnIBD) diagnosed after solid organ transplant (SOT) are not well-described, particularly since the advent of biologic therapy for treatment of IBD.

**Methods:**

We conducted a single-center, retrospective review of SOT recipients between 2010 and 2022 at the University of Minnesota Medical Center who were diagnosed with IBD after transplant.

**Results:**

Of 89 patients at our center with IBD and a history of SOT, five (5.6%) patients were diagnosed with IBD post-transplant (three liver, one kidney, and one simultaneous liver and kidney): three patients were female and four were Caucasian. Mean age at transplant and IBD diagnosis were 46.7 and 49.4 years respectively. Indication for transplant were alcohol-related cirrhosis (*n* = 2), idiopathic fulminant hepatic failure (*n* = 1), metabolic dysfunction-associated steatotic liver disease (*n* = 1), and IgA nephropathy (*n* = 1). Four patients were diagnosed with ulcerative colitis (UC) and one with Crohn's disease (CD). Three patients (all with UC) required escalation to a biologic therapy. Four patients were in clinical remission from IBD at last follow-up, one patient required IBD surgery, while there was no rejection and no deaths following IBD diagnosis.

**Conclusion:**

dnIBD post-SOT is uncommon, while newer IBD therapies may be safe and effective. Further study is required to better understand the natural history and IBD outcomes of this population relative to non-SOT patients.

## Introduction

Inflammatory bowel disease (IBD) is a chronic, inflammatory condition of the gastrointestinal tract (GI), which includes ulcerative colitis (UC) and Crohn's disease (CD) (1). IBD's pathophysiology remains poorly understood but it is likely due to complex interactions between environmental factors, genetics, immune responses, and the microbiota (2). Solid organ transplantation (SOT) in patients with IBD is uncommon but those with primary sclerosing cholangitis (PSC) have slow progression to end-stage-liver-disease, with a median duration of time to liver transplantation (LT) of 15–20 years post diagnosis (3, 4).

Lifelong immunosuppression is required to prevent organ rejection after SOT; therefore, the development of de-novo (new onset) IBD (dnIBD) post SOT seems counterintuitive with immunosuppression use. Standard immunosuppression post SOT includes calcineurin inhibitors, which have a variable relationship with IBD. Tacrolimus, a calcineurin inhibitor, is associated with worsening IBD disease activity in pre-existing IBD (5, 6). Conversely, tacrolimus has been used as treatment for CD. Additionally, cyclosporine, is used as salvage therapy in cases of severe IBD refractory to intravenous steroids in hospitalized patients (7). However, dnIBD post SOT has been described, albeit with most cases following LT (8, 9). A recent case series from Japan described six cases of dnIBD in a cohort of patients who underwent living donor kidney transplantation (10).

In 1998, the FDA approved infliximab, a monoclonal antibody targeting tumor necrosis factor-alpha (TNF-α), for treatment of CD. The introduction of biologic therapy has transformed clinical outcomes for patients with IBD with higher rates of remission and a reduction in surgical interventions, while also reducing hospitalization rates and enhancing patients' quality of life (11, 12). Biologic therapies are integral to the treatment of moderate to severe IBD, with current guidelines recommending their use as first-line treatment particularly in the era of top-down approach to therapy for IBD (12). A recent meta-analysis reported that biologic and small molecule therapies appear to be well-tolerated in SOT patients with IBD, although data on the use of biologic therapy in patients with dnIBD after SOT are limited (13).

At this time, there is no clear consensus on management or surveillance of dnIBD after SOT, particularly surrounding the use of biologic therapy. In fact, diagnosis of dnIBD in SOT patients is challenging given the broad differential diagnosis, specifically infection and medication side effects. The aim of this study was to characterize the clinical presentation, management and clinical outcomes of individuals developing IBD after SOT at our institution over a period when the use of biologic therapy for non-SOT IBD was well-established.

## Methods

We conducted a retrospective review of the electronic medical records of patients who underwent SOT at University of Minnesota Medical Center from 1/1/2010 through 12/31/2022. Patients were categorized with dnIBD if they developed IBD during any period after SOT, without a pre-SOT IBD diagnosis. Individuals with clinical signs or endoscopic evidence of IBD prior to SOT were excluded; four out of five patients included in the study had a normal colonoscopy prior to transplant. Records were reviewed for demographic information, SOT indication and outcomes, and IBD diagnosis and outcomes. Clinical documentation was reviewed for presenting symptoms and severity at time of IBD diagnosis, as described by the treating physicians' overall impression. Objective disease activity scores obtained from clinical documentation and endoscopy reports were reviewed when available. Response to therapy was determined by the treating provider's documented impression. Individuals with SOT and other forms of colitis who did not meet criteria for IBD were excluded from the study. Infection was ruled out in all cases at the time of diagnosis with negative clostridium difficile polymerase chain reaction (PCR) and enteric pathogen panel (when available), or negative stool bacterial culture, and ova and parasites testing. This study was conducted with the approval of the University of Minnesota Institutional Review Board (STUDY00017400).

## Results

### Patient characteristics

Eighty-nine patients with a history of SOT and a diagnosis of IBD were initially identified during the study period: five patients were diagnosed with IBD after SOT and included in the study. Three (60%) patients were women. The mean age at transplant was 46.7 years (range, 24–66 years), mean age at IBD diagnosis was 49.4 years (range, 27–68 years), and the mean time from SOT to diagnosis of IBD was 3.1 years (range, 3–3.75 years). Four (80%) patients were non-Hispanic white, and one patient was Black/African American (Table 1). Four (80%) patients have no family history of IBD, with one patient having a family history of CD.

**Table 1:** Demographic and clinical characteristics of cohort.

Patient	Sex	Race	Smoker?	Type of SOT	Age at SOT (years)	Indication for SOT	IBD type	Disease distribution	Age at IBD diagnosis (years	IBD symptoms at diagnosis	Time from transplant to diagnosis (years)	Immunosuppression at diagnosis	IBD medications at last follow-up	Disease status at last follow-up
1	M	Caucasian	Never	Liver	50	ETOH cirrhosis	UC	Procto-colitis	54	Hematochezia, Tenesmus	3.75	Tacrolimus	Vedolizumab, mesalamine	Clinical remission, Mild on endoscopy
2	F	Black/African American	Never	Liver	32	Autoimmune hepatitis	UC	Pan-colitis	35	Hematochezia	2.5	Tacrolimus	Ozanimod	Active flare, Moderate on endoscopy
3	F	Caucasian	Never	Liver	60	Acute liver failure	UC	Pan-colitis	63	Diarrhea, weight loss	3	MPA and prednisone	Mesalamine	Clinical and endoscopic remission
4	F	Caucasian	Never	Kidney	24	IgA nephropathy	UC	Pan-colitis	27	Hematochezia, weight loss	3.25	Tacrolimus and MMF	Vedolizumab	Clinical and endoscopic remission
5	M	Caucasian	Former	Liver and Kidney	66	MASLD cirrhosis	CD	Ileo-colonic	68	Diarrhea, weight loss	3	Tacrolimus and Azathioprine	Azathioprine	Clinical and endoscopic remission

CD, Crohn's disease; ETOH, alcohol; IBD, inflammatory bowel disease; MASLD, metabolic dysfunction-associated steatotic liver disease; MMF, mycophenolate mofetil; MPA, mycophenolic acid; UC, ulcerative colitis.

### SOT characteristics

Three (60%) patients received LT, one received kidney transplant (KT), and one received simultaneous liver-kidney (SLK) transplant. Indications for SOT included IgA nephropathy, alcohol-related liver disease, and metabolic dysfunction-associated steatotic liver disease (MASLD). No patient had a history of transplant rejection prior to diagnosis of IBD. Four (80%) patients were taking tacrolimus-based immunosuppression regimens at the time of diagnosis of IBD, one patient (20%) was taking mycophenolate mofetil (MMF) in addition to tacrolimus, and another patient (20%) was taking mycophenolic acid (MPA) and low dose prednisone (5 mg per day) (Table 1).

### IBD characteristics

#### Presentation

**Figure 1:**
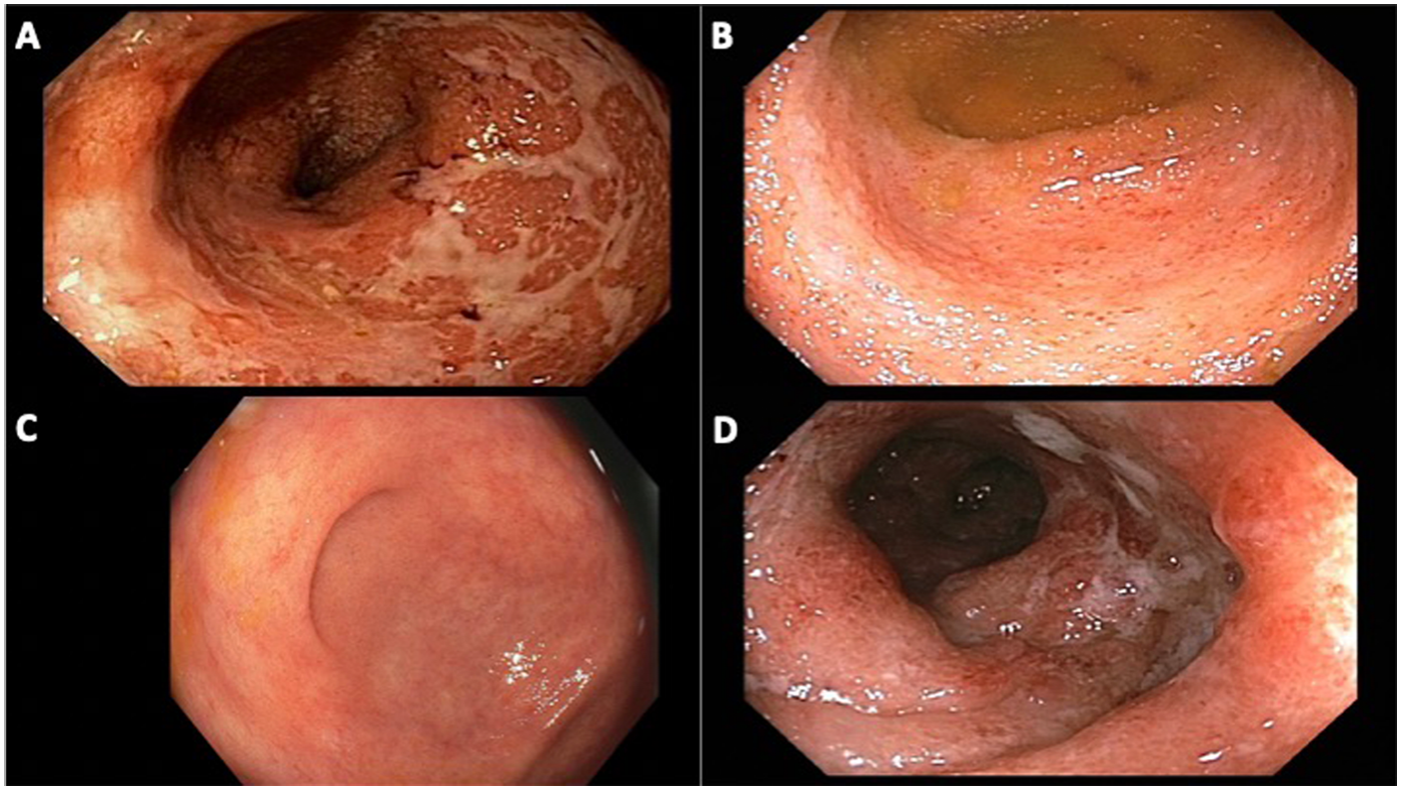
Endoscopic images of patients from our cohort with ulcerative colitis. **(A)** patient 1, rectum with diffuse erythema and congestion, ulcerated mucosa, at time of diagnosis of ulcerative proctocolitis; **(B)** patient 2, cecum with congestion, erosions and friability, at time of diagnosis of ulcerative pan-colitis; **(C)** patient 3, cecum with minimal patchy erythema and granularity, on oral mesalamine for 18 months after diagnosis of ulcerative pancolitis; **(D)** patient 4, sigmoid colon with diffuse edema, patchy erythema and ulcerations, at time of diagnosis of ulcerative pan-colitis.

Four (80%) patients presented with hematochezia, while the other patient presented with diarrhea. Four (80%) patients were diagnosed with UC and one with CD. Two (40%) patients presented with severe disease based on the treating providers' impressions of endoscopic Mayo score of 3.^7^ Three (75%) patients with UC presented with ulcerative pancolitis. The patient with CD presented with diarrhea and abdominal pain and was diagnosed with ileal CD complicated by a stricture (Table 1). Endoscopic and histopathologic features were consistent with IBD in all patients (Figures 1A, B, D).

**Figure 2:**
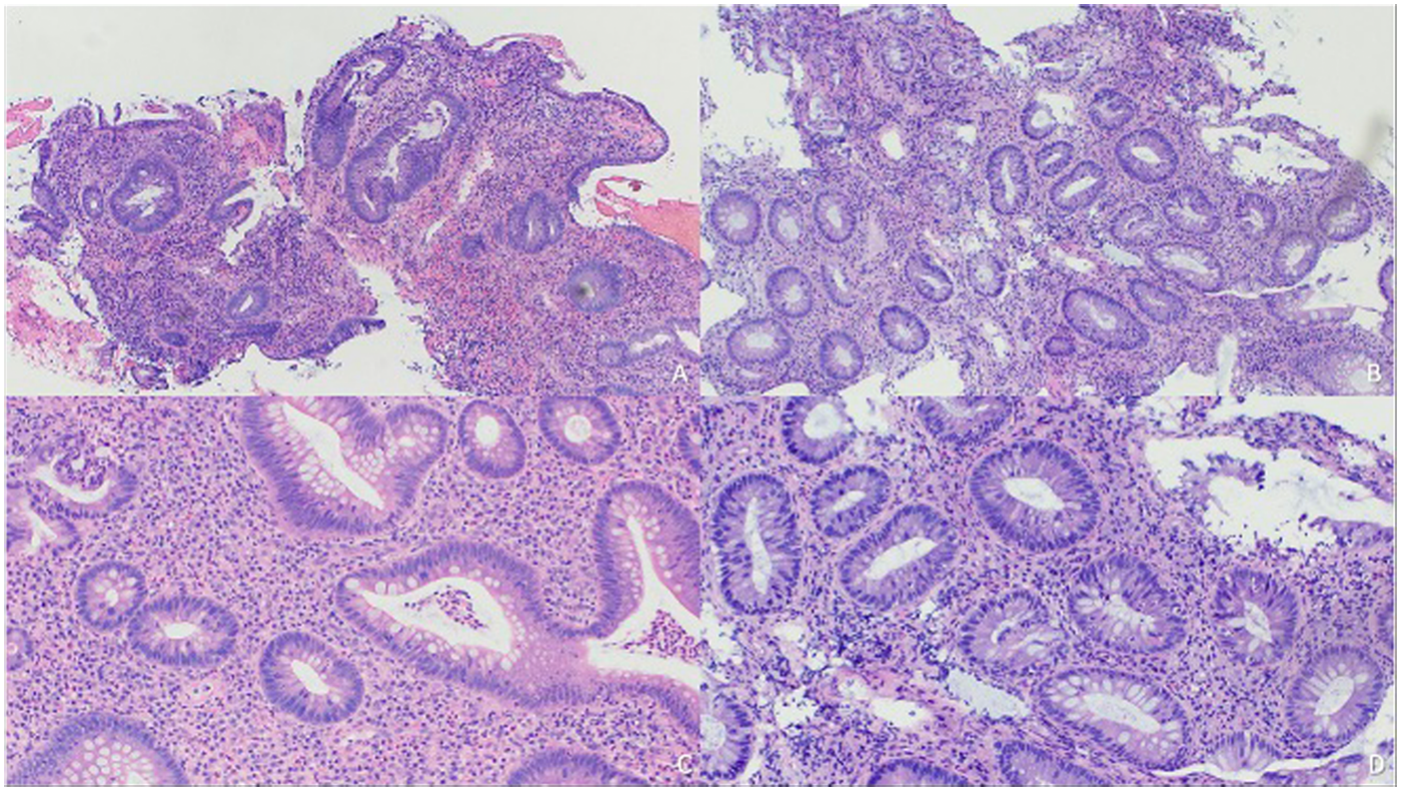
Pathology images of patients from our cohort with ulcerative colitis. H&E staining showing marked lamina propria lymphoplasmacytic inflammation, irregular crypt, and neutrophilic cryptitis and crypt abscess. **(A)** 50X. **(B)** 100X. **(C)** 200X. **(D)** 200X.

Two patients were on MMF and MPA at the time of diagnosis, which are known to induce IBD-like inflammation. Histologic features of MMF/MPA-induced colitis, such as apoptotic bodies and eosinophilic infiltrates, were not seen (Figure 2). Both cases underwent expert pathology review and were deemed consistent with IBD rather than drug-induced colitis. In both cases, discontinuation of MMF/MPA was considered if no improvement was observed following IBD treatment.

#### Management within first 6 months of IBD diagnosis

Within the first 6-months of diagnosis, three individuals required escalation to an advanced therapy with vedolizumab. Two patients, both with severe UC, were treated with corticosteroids at the time of diagnosis. By six months, both transitioned to vedolizumab: one experienced clinical remission, while the other did not and was subsequently transitioned to ustekinumab.

Two patients with mild UC were initially treated with 5-aminosalicylic acid (ASA). One experienced clinical remission on mesalamine monotherapy while the other had ongoing disease activity and was transitioned to vedolizumab. This patient experienced clinical remission on vedolizumab.

The patient with CD continued treatment with azathioprine, which was already being used as immunosuppression for his LT. No dose adjustments were made to azathioprine. This patient was documented as being in clinical remission at 6-months post-diagnosis.

#### Management beyond 6-months of IBD diagnosis

The patient with CD underwent right hemicolectomy at ∼12 months after IBD diagnosis. Ileal biopsies taken at a surveillance colonoscopy showed evidence of a villous adenoma and a possible adenocarcinoma. Histopathology of the resection specimen showed two synchronous ileal adenocarcinomas- one adenocarcinoma was poorly differentiated and the other was moderately differentiated. Azathioprine was continued at the same dose post-ileal resection and the patient was in full remission at last follow-up.

At the time of last follow-up, three of the patients with UC were in clinical remission: one on mesalamine monotherapy and two on vedolizumab (Figure 1C). Two UC patients in clinical remission were also in endoscopic and histologic remission. The patient with UC on vedolizumab and mesalamine had mild inflammation on endoscopy. The final patient, who had been transitioned to ustekinumab within the first 6 months of IBD diagnosis, had poorly controlled disease at the time of last follow-up and was initiating ozanimod (Table 1).

#### Disease complications

One patient with UC, who was maintained on mesalamine, developed recurrent clostridium difficile infection since IBD diagnosis and received fecal microbiota transplant. The patient with CD developed two ileal adenocarcinomas (see above), but no dysplasia was noted in the patients with UC. No patients developed acute organ rejection, however, one patient developed graft failure due to chronic antibody-mediated rejection and recurrent IgA nephropathy. Transplant immunosuppression medications were not changed over the course of IBD therapy. There were no patients with documented extraintestinal disease after IBD diagnosis and no patients had died by the time of last follow-up.

## Discussion

In this report, we describe our experience with dnIBD following SOT, which accounted for 5.6% of our center's IBD-SOT population. Clinical presentations of dnIBD were similar to those in non-SOT IBD, with MMF/MPA-associated colitis considered in two patients. Most SOT patients with dnIBD at our center required an advanced therapy, which favored biologic agents with preferable safety profiles. Importantly, no major treatment complications were noted in our patients.

### Pathophysiology of IBD

The pathophysiology of IBD remains incompletely understood, but likely involves a variety of factors including genetics, environmental, immune responses, and microbes (2, 14). Genome studies have identified more than 200 genetic mutations that have been linked to IBD (15). T-cells of the adaptive immune response have been implicated in the development of IBD, as these responses are heightened in UC and CD (15). Almost paradoxically, immunosuppression in SOT patients is primarily directed at regulation of the alloimmune T-cell response to an allograft (15). Of note, most of the patients in our cohort did not have immune-related indications for transplantation, which can be associated with development of IBD.

Other factors such as medication use and the microbiome, are also important in the development of *de novo* IBD. The use of medications such as statins and non-steroidal anti-inflammatory drugs (NSAIDs), have been associated with an increased risk of developing IBD (16). Transplantation results in changes to a patient's microbiome, while the use of immunosuppression itself leads to immune responses against the gut microbiome that are dysregulated, which increases the risk of IBD development (17). In our cohort, one patient had a history of IgA nephropathy, which is associated with IBD, although the exact relationship (and pathophysiology) remains poorly understood (18–20). Finally, smoking has been implicated with the development of IBD, particularly CD- the only patient in our cohort with CD was a former smoker (21).

### Differentiation from drug-induced colitis

MMF-induced colitis is an important consideration in SOT patients presenting with diarrhea and/or hematochezia. Two patients in our cohort were taking either MMF or MPA at the time of diagnosis of IBD. MMF toxicity can affect the entire GI tract, while MMF-induced colitis has been reported in up to 9% of SOT recipients on MMF who undergo colonoscopy (22, 23). Histological findings of MMF-induced colitis may be clinically and endoscopically indistinguishable from IBD, although histology shows a predominance of eosinophils in the mucosa with a lack of apoptotic micro-abscesses and endocrine cell aggregates in the lamina propria (5, 22). Discontinuation of MMF may cause a quick resolution of symptoms, but symptoms can persist for months in specific cases, raising the concern for IBD (5, 23). In both of our cases who were taking MMF/MPA, drug-induced colitis was considered, but a satisfactory diagnosis of IBD was made after pathology review and close observation over time. This highlights the importance of multidisciplinary teams including a transplant hepatologist, gastrointestinal pathologist and IBD specialist working together to balance the safety of changing SOT medications while treating intestinal inflammation (23).

### Biologic therapy in SOT patients with dnIBD

Our study provides necessary granular detail on the use of biologic therapy in SOT patients with dnIBD. In particular, this level of detail can provide important context to clinicians needing to make decisions on the use advanced therapies in patients with more severe disease. There is currently limited data on the use and efficacy of advanced therapies in SOT patients with dnIBD (24, 25). A recent meta-analysis provided minimal safety information on SOT patients with dnIBD who received biologic therapies (13). In general, the use of biologic therapy for all indications in SOT recipients is safe and effective, albeit with a concern for the development of severe infections (24). Biologic and small molecule therapies in SOT patients with pre-existing IBD are largely well-tolerated: a meta-analysis showed that infectious complications were similar to rates seen in SOT patients without IBD, as were rates of colectomy and discontinuation of biologic therapy (13).

In our cohort, vedolizumab was the most used biologic agent. Vedolizumab is a gut-selective, anti-integrin blocker that binds to leucocyte integrin α4β7 that is considered to have a lesser effect on the immune system when compared to other monoclonal antibodies, making it a preferable medication for SOT recipients (26). A recent systematic review and meta-analysis showed that patients with UC on vedolizumab had a lower risk of serious infections when compared to infliximab, although another study reported that infliximab may be more effective at induction of remission in these patients (27, 28). However, a systematic review and meta-analysis evaluating outcomes of biologic and small molecule therapies, specifically in SOT patients with major emphasis of a diagnosis of IBD pre-transplant, suggested that rates of severe infections were higher in patients taking vedolizumab, albeit the studies used were not adjusted for disease severity and other confounders (13). Only one patient on systemic immunosuppression for IBD in our cohort developed a serious or severe infection. Our experience suggests that providers may prioritize (perceived) safety when selecting an advanced therapy. Furthermore, transplant immunosuppression regimens were stable in our cohort: we hypothesize that treating gastroenterologists may have deferred to transplant immunosuppression as “primary immunosuppression” and subsequently chosen milder IBD treatments. Qualitative studies examining therapeutic relationships between transplant and gastroenterology providers are needed to better understand decision-making regarding immunosuppression in this patient population.

### Clinical outcomes of patients with dnIBD after SOT

Our cohort adds to the literature by describing outcomes of IBD development post SOT. It is possible that patients who develop IBD while taking SOT immunosuppression may have a more aggressive underlying disease phenotype, however clinical outcomes in our cohort were favorable as four of five patients achieved clinical remission at the time of last follow-up. A multicenter retrospective study of SOT in an pre-existing IBD population described a clinical remission rate of 61% in patients following transplantation (29). One patient in our cohort did require surgery for a malignant ileal stricture. In non-SOT patients with CD, rates of surgery are decreasing, possibly related to advances in medical therapy (30, 31). In two studies of patients with pre-existing IBD who underwent SOT, rates of surgery were low both before and after transplantation (29, 32).

Of note, no patients in our cohort developed acute rejection following diagnosis of dnIBD over the follow-up period. As mentioned previously, transplant immunosuppression medication regimens were stable over the follow-up period. Treatments for IBD primarily target T-cell activity in the immune system, while activation of the T-cell response is one of the main cascades responsible for the development of acute rejection (33). It is possible that the presence of IBD may in fact be a protective factor against rejection in SOT patients: acute rejection was also rare in a cohort of patients with pre-existing IBD who underwent non-LT SOT (32).

### Study limitations

Our study has clear limitations related to both the retrospective nature of the data collection and the small sample size. The retrospective study design leads to variable time follow-up and limits the ability to address confounding factors: specifically, we cannot be 100% sure that there was no IBD preceding SOT. However, medical records were extensively manually reviewed and there was, at a minimum, no clinical IBD activity. As a retrospective analysis, there is potentially important data on risk factors, e.g., HLA typing, that is not available for our review. Additionally, the small sample size limits the ability to infer accurate trends among this population but does allow for data collection in more granular detail. Larger sample sizes are clearly needed to accurately estimate the true rate of clinical outcomes in this unique patient population. Additionally, as a single center study, the results may not be generalizable, but we anticipate that most transplant centers reflect a population similar to ours. These are unavoidable limitations of this analysis. However, our study provides information to guide future studies, which are required to better understand the natural history and outcomes of dnIBD after SOT.

In summary, we report our experience of five patients who developed IBD following SOT. dnIBD post SOT is uncommon but should be considered in SOT patients presenting with typical IBD symptoms. The key highlight of our small study is that most individuals required specific treatment of IBD in the post-transplant setting, with some requiring an advanced therapy to achieve clinical remission. And, when choosing IBD therapy, there appears to be a provider bias towards medications with a favorable safety profile. Multicenter studies are required better understand clinical outcomes, impact of transplant type, and long-term impact of biologic therapies in patients with dnIBD after SOT.

## Data Availability

The raw data supporting the conclusions of this article will be made available by the authors, without undue reservation.
